# Assessment of the efficiency of the routine epidemiological surveillance system for malaria at the Tambacounda Health District, Senegal

**DOI:** 10.11604/pamj.2024.48.41.41888

**Published:** 2024-06-04

**Authors:** Tidiane Gadiaga, Mouhamadou Faly Ba, Siré Sagna, Bayal Cissé, Doudou Sène, Samba Cor Sarr, Babacar Gueye, Sylla Thiam, Elhadji Ba Konko Ciré, Jean Louis Abdou Ndiaye

**Affiliations:** 1 Planning, Research and Statistics Department, Ministry of Health and Social Action, Fann-Dakar, Senegal,; 2 Health and Development Institute, Cheikh Anta Diop University, Fann-Dakar, Senegal,; 3 Tambacounda Health District, Ministry of Health and Social Action, Fann-Dakar, Senegal,; 4 National Malaria Control Program (NMCP), Ministry of Health and Social Action, Fann-Dakar, Senegal,; 5 Iba Der Thiam University, Université of Thies, Thies, Senegal

**Keywords:** Epidemiological surveillance, malaria, Tambacounda, Senegal

## Abstract

**Introduction:**

as part of the fight against malaria, epidemiological surveillance (ES) is one of the key pillars of the global technical strategy 2016-2030 to combat this disease. However, in the south-east of Senegal, where malaria poses a major public health problem, epidemiological surveillance has until recently been very neglected. To help reduce malaria-related morbidity and mortality in Senegal, an evaluation of the routine malaria ES system was conducted in the Tambacounda Health District in 2021.

**Methods:**

we conducted a cross-sectional, descriptive survey of 27 health structures in Tambacounda district from 20^th^ February to 1^st^ March 2022.

**Results:**

overall, the routine ES system in the district was acceptable according to its users, with satisfactory tool filling time in 96.3% of the structures in our study, a cumulative completeness of reports at 92% despite a 77% promptness. The data collected at the services delivery points (SDP) level allowed a representativeness of the ES system in 100% of health facilities. The ES system was also rated as simple by 74.1% of SDP managers even though only 55.6% of them were trained. However, the stability of the system was low because 55.6% of SDP had staff to ensure the continuity of ES service despite the availability of management tools (100%) and the telephone network (96.3%). The same was true for the usefulness of the ES because only 25.9% of SDP managers analyzed their produced data. The reported ES and malaria morbidity data were not adequate. On the other hand, the ES system was reactive with a speed of transmission of information at 96.3% and a possibility of rapid diagnosis and management of cases at 100%.

**Conclusion:**

the routine malaria ES system at the level of health facilities in Tambacounda District was acceptable, simple, flexible, representative, and responsive. However, an increase in qualified staff at the health posts, capacity strengthening of the ES staff and regular supervision of SDP are needed essentials to make the district's malaria surveillance system more efficient.

## Introduction

Epidemiological surveillance (ES) is a continuous, systematic process of collecting, analyzing, and interpreting data on specific health events, important for the planning, implementation, and evaluation of public health practices, closely associated with their fair dissemination to those who need to be informed [1]. It monitors the health system and integrates communicable diseases that are the most common causes of death and disability in the African Region. These diseases pose a significant threat to the well-being of African communities, while the control and prevention interventions available to combat them are well known. Among them, malaria occupies an important place. According to the World Health Organization (WHO), 247 million cases of malaria were recorded worldwide in 2021 causing 619,000 deaths, most of which are children under 5 years of age in sub-Saharan Africa [2]. As part of the fight against malaria, epidemiological surveillance is one of the pillars of the global technical strategy 2016-2030 to combat this disease. In Senegal, malaria is one of the main causes of death among children below the age of five years and remains the most common cause of fever among this age group.

For a population of 17,316,448 inhabitants in 2022, the malaria incidence was 48.0% or 831,514 confirmed malaria cases [3], which were unevenly distributed across the 14 regions of the country. The regions of Kolda, Tambacounda, and Kedougou alone bear the bulk of the malaria burden with 83.3% of malaria cases while they cover only 11.3% of Senegal's population [3]. Despite the implementation of several malaria control interventions [4] (distribution of long-acting impregnated mosquito nets, raising awareness on the use of mosquito nets, destruction of mosquito breeding sites, intermittent preventive treatment of malaria in pregnant women with sulfadoxine-pyrimethamine, chemo prevention of seasonal malaria in children from 43 to 120 months with sulfadoxine-pyrimethamine and amodiaquine, free diagnosis and management of malaria cases, management of malaria cases at home), this disease remains a national priority especially for the regions of Kolda, Tambacounda and Kedougou. The same is true for the Health District of Tambacounda where malaria is a priority disease with incidences that increased from 161.4% to 186.3% between 2019 and 2020 [3].

As part of routine epidemiological surveillance of malaria, malaria-related data are collected weekly by all health districts in the country through health facilities and transmitted to the national level through weekly entry into the District Health Information Software (DHIS2) platform. This ES data guided health workers in decision-making to implement appropriate control strategies and prevention activities [5]. Despite the importance of this tool in the fight against malaria, studies on the acceptability and efficiency of the epidemiological surveillance system of malaria are scarce in Senegal. As a result, opportunities for corrective measures, appropriate responses to malaria, and saving lives are lost [6].

We therefore conducted this study to assess the efficiency of the routine epidemiological surveillance system for malaria. The specific objectives of this study were to measure the completeness and timeliness of ES data, to describe the weekly trend of malaria cases, to assess the attributes of the malaria ES system, and to determine the strengths, weaknesses, opportunities, and threats of the Tambacounda District Malaria ES system.

## Methods

**Study framework:** the Health District (HD) of Tambacounda is located in the department and region of the same name. The region of Tambacounda, located in the south-east of the country is the largest region and concentrates only 5.5% of the population making it the region with the lowest occupation of space.

The Health District of Tambacounda has an area of 11,416 km^2^ with an estimated population in 2021 of 305,801 inhabitants, a density of 27 inhabitants/km^2^ and a natural increase rate of 2.7%. It consists of 26 health posts and 1 reference health center. It also includes five secondary health centers, one public type 2 health facility, five private clinics, four private practices, 37 health huts, and 294 home care providers (HCP) who submitted their surveillance data at the level of the coordinating health facilities. At the district level, all health facilities carry out routine epidemiological surveillance of malaria by reporting a circuit of transmission of information. The SDP officers compile data from the various health facilities in their catchment areas and then transmit them to the district malaria focal point. They also ensure the entry of this data every Monday before noon in the District Health Information Software (DHIS2) platform.

**Type and period of study:** this was a cross-sectional survey of the routine epidemiological surveillance system for malaria at the Tambacounda Health District. Data were collected from 20^th^ February to 1^st^ March 2022. It covers the period from the first week (W1) to the 53^rd^ week (W53) in 2021.

### Study population and sampling protocol

**Study population:** these were the service delivery points (SDP) of the Tambacounda Health District (HD).

**Inclusion criteria:** included in the study were all Tambacounda Health District SDP who conducted routine epidemiological surveillance of malaria from week 1 to week 53 of 2021.

**Non-inclusion criteria:** not included in the study were any SDP whose manager was not available to participate in this work for one reason or another.

**Sampling:** the selection was exhaustive. These were the 27 health structures with an area of responsibility (26 health posts and 1 reference health center) of the HD of Tambacounda.

### Data collection

**Collection tool and data sources:** a questionnaire developed based on the models recommended by the WHO [7] was used for the survey. This questionnaire includes items such as demographic information, case identification, data recording and reporting, data analysis, investigation, and preparedness for the outbreak response. This questionnaire was used to assess attributes such as simplicity, usefulness, flexibility, stability, and acceptability.

Consultation logs and weekly and monthly reports at the SDP level were used to assess data quality and also to study attributes such as data adequacy, representativeness, and responsiveness. The DHIS2 platform was used as another data source to determine completeness, timeliness, and number of registered malaria cases. The data was extracted from the DHIS2 platform on 20 February 2022 at 15:55.

**Collection method:** among the members of the district management team, two investigators who had basic training in epidemiological surveillance and malariology were selected and trained on the administration of the questionnaire. They were accompanied by the malaria focal point of the Tambacounda medical region. The orientation session took place on 18^th^ February 2022. Thereafter, the collection was made in ten (10) days from February 20^th^ to March 1^st^, 2022. After obtaining free and informed consent from SDP officials, the data was collected using: a consultation held with the managers of the health facilities through a questionnaire to collect their assessment of the ES system in relation to its strengths, weaknesses, opportunities, and threats (SWOT). The responses of the SDP managers, which were similar, were synthesized and classified according to the heading’s strengths, weaknesses, opportunities, or threats. Verification of on-site data was performed by the investigators to assess the accuracy of the data transmitted. Inspection of the DHIS2 platform was also conducted to assess the completeness, promptness, and trends of malaria cases reported during the study period.

**Operational definition of variables:** the effectiveness of the routine epidemiological surveillance system was assessed through the following variables:

**Completeness:** this is the peak of the number of reports received (numerator) over the number of reports expected (denominator), over a period of time.

**Promptness:** this is the percentage of the number of reports that were received on time (by the deadline set by the district in consultation with claimants) to the numerator on the number of reports expected at the denominator.

**Usefulness:** it is the capacity for the ES system to achieve the objectives set (detect trends, identify populations at risk, monitor the impact of the program or interventions, alert early to health problems) and take action.

**Simplicity:** it will be assessed based on the availability of monitoring guidelines, the ease of use of case definitions, SE management tools, and feedback.

**Flexibility:** adapting the system when introducing a change in monitoring (new variables or data sources).

**Representativeness:** this is the possibility of describing malaria cases according to the characteristics of the population in terms of time and people.

**Acceptability:** it is appreciated in relation to the time required for the completion of forms, the completeness of reports, and the prompt transmission of reports.

**Responsiveness:** this is the necessary step in the transmission of information from one level of the surveillance system to another and the possibility of diagnosis and rapid case management.

**Stability:** this is the absence of interruption of monitoring activities (availability of tools, absence of network during data transmission, availability of qualified human resources for continuity of service).

**Data quality:** It will be assessed based on the proportion of missing data, proportion of reporting errors, and supervision of SDP on on-site data quality.

### Data analysis

**Data entry:** the collected data was entered into the Epi Info software version 7.2. Controls were integrated during the preparation of the input mask to limit input errors. A cleaning of the seized files was done with the software’s analysis program and corrected the outliers.

**Methods of analysis:** the analysis of the data was done in a descriptive study with a univariate analysis that had made it possible to determine the position parameters of the quantitative (mean, standard deviation) and qualitative (absolute, relative frequencies) variables. The Strengths, Weaknesses, Opportunities, and Threats (SWOT) of the Tambacounda HS in the context of the Malaria SE have been identified.

**Ethics:** participation in this study was voluntary. It was done after free and informed consent. The evaluators administered a complete information form to the person in charge of the health structure. Confidentiality and anonymity were respected throughout the process. Apart from the completeness and promptness that were available in the national DHIS2 platform, the name of the SDP was hidden in the analysis of the other attributes of the ES. The study received authorization from the Directorate of Planning, Research, and Statistics of the Ministry of Health and Social Action of Senegal.

## Results

Twenty-seven health facilities were surveyed with a response rate of 100%. Of these, 44.4% were headed by nursing assistants, 37.1% by state-enrolled nurses, 14.8% by midwives, and only 3.7% by a doctor. These SDPs covered an average of 19.1 villages and/or neighborhoods with a standard deviation of 18.2. There was an average of 1.3 health boxes per area of responsibility SDP. Sites for the management of malaria cases at home (HCP) averaged 10.9 per SDP.

**Acceptability:** overall, the malaria ES system was acceptable according to users in the health facilities of the Tambacounda Health District, with the time taken to fill in the management tools being acceptable in 96.3% of cases. There was also good completeness of the reports in 85.2% of cases; even if promptness was good in only 51.9% of The SDP (Table 1).

**Table 1:** distribution of responses to the “acceptability of the epidemiological surveillance (ES) system” attribute according to the Tambacounda Health District (HD) services delivery points (SDP) and distribution of SDP performance according to the items of the representativeness attribute of the ES to the Tambacounda HD in 2021

Parameters	n	Percentage
**Time spent filling tools**		
Acceptable	26	96.3
Not acceptable	1	3.7
**SDP with cumulative completeness above 80%**		
Yes	23	85.2
No	4	14.8
**SDP with cumulative promptness above 80%**		
Yes	14	51.9
No	13	48.1
**Possibility of describing cases in time, place, and persons**		
Yes	27	100
No	0	0
**Representation of cases on a graph**		
Yes	9	33.3
No	18	66.7

**Completeness:** for the 27 health facilities in our survey, the cumulative completeness was on average 92%. Only 4 SDPs had a completeness of less than 80%.

**Promptness:** for this indicator, 51.8% of SDP were at a cumulative speed of over 80%. Only one of the SDP (3.7%) had 100% promptitude throughout the year. Among the 27 SDP, 3 of them (11.1%) had cumulative prompts of less than 50%.

**Figure 1:**
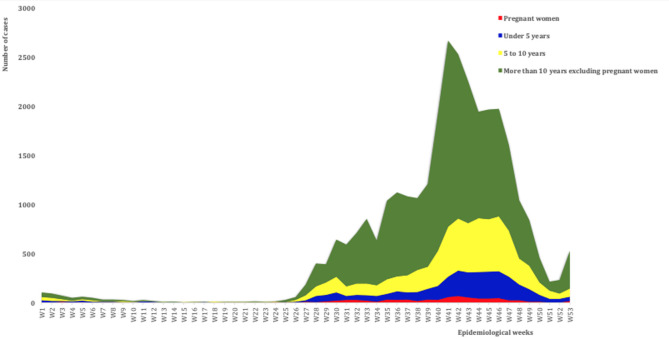
weekly evolution of malaria cases reported by age group and Tambacounda Health District (HD) status from W1 to W53 in 2021

**Representativeness:** all 27 health facilities surveyed had the same collection and reporting tools taking into account localities, human characteristics (age, sex), and period. Thus, it was possible to represent all malaria cases according to time, place, and people in 100% of the SDP. However, only 33.3% of the structures represented malaria cases on graphs (Table 1). In 2021, the district’s 27 SDP reported 30,846 malaria cases with 6 deaths. Between W12 to W25, regardless of age group or status, only a few sporadic cases of malaria are recorded per week at the district level, corresponding to the period of low transmission. The age group over 10 years was the most representative with a total of 19,851 cases of malaria and a peak at W41 with 1,890 cases. For children under 5 years of age and those aged 5 to 10 years, the total number of malaria cases was 3,385 cases and 6,871 cases with peaks at W46 respectively (Figure 1). In pregnant women, 739 cases of malaria were reported during 2021.

**Simplicity, stability, and flexibility of malaria epidemiological surveillance:** epidemiological surveillance guidelines were only available in 14.8% of the SDP surveys. Providers felt that case definitions for malaria ES were simple (74.1%) even though only 55.6% of SDP officials were trained in ES (Table 2).

**Table 2:** distribution of parameters related to the simplicity, stability, and utility attributes of malaria epidemiological surveillance (ES) in the Tambacounda Health District (HD) in 2021

Attributes	Parameters	n	Percentage
**Simplicity**	**Availability of ES directives**		
	Yes	4	14.8
	No	23	85.2
	**Case definition view or circular note**		
	Yes	22	81.5
	No	5	18.5
	**Use of case definitions for malaria ES**		
	Yes	21	77.8
	No	6	22.2
	**Simple case definitions**		
	Yes	20	74.1
	No	7	25.9
	**Easy-to-use monitoring management tools**		
	Yes	20	74.1
	No	7	25.9
	**Training of the SDP manager on malaria ES**		
	Yes	15	55.6
	No	12	44.4
	**Feedback or feedback malaria from the district**		
	Yes	23	85.2
	No	4	14.8
**Stability**	**Non-breaking ES management tools**		
	Yes	27	100
	No	0	0
	**Network availability for data transmission**		
	Yes	26	96.3
	No	1	3.7
	**Availability of a lining for the continuity of the ES**		
	Yes	12	44.4
	No	15	55.6
**Flexibility**	**Consideration of malaria diagnosis in tools**		
	Yes	27	100
	No	0	0
	**Tools adapted to malaria ES guidelines**		
	Yes	27	100
	No	0	0
	**Possibility of change of management tools/variable introduction**		
	Yes	27	100
	No	0	0

SDP: services delivery points

The management tools were easy to use with items adapted to malaria ES guidelines. In all the SDP surveyed (100%), there was the rapid diagnostic test (RDT) column in the general consultation registers, so a possibility of considering the diagnosis of malaria in the tools. Even if there was an availability of ES management tools and a telephone network in health facilities, the stability of the ES was problematic because in 55.6% of health facilities, if the head nurse (HN) was absent, there were no more qualified personnel to ensure the continuity of the service in a correct way.

**Usefulness of the epidemiological surveillance system:** there was a misperception of the usefulness of the ES. Of the providers surveyed, only 25.9% of them analyzed the SE data produced on-site. Due to ignorance of the usefulness of ES, malaria case trend lines were not up to date in 85.2% of SDP and only 14.8% of SDP managers compared their data with those of neighboring SDP for early warning (Table 3).

**Table 3:** distribution of services delivery points (SDP) performance by assessed parameters of the ‘’usefulness’’ and “reactivity’’ attribute of the malaria epidemiological surveillance (ES) system at the Tambacounda Health District (HD) in 2021

Attributes	Parameters	n	Percentage
**Usefulness**	**Management of suspected and/or confirmed cases of malaria**		
	Yes	27	100
	No	0	0
	**Analysis of on-site data**		
	Yes	7	25.9
	No	20	74.1
	**Identification of possible causes of problems**		
	Yes	9	33.3
	No	18	66.7
	**Analysis of trends in malaria cases**		
	Yes	7	25.9
	No	20	74.1
	**Up-to-date trend lines**		
	Yes	4	14.8
	No	23	85.2
	**Comparing data with a neighboring SDP for alert**		
	Yes	4	14.8
	No	23	85.2
**Reactivity**	**Timeliness of information transmission to HD**		
	Yes	26	96.3
	No	1	3.7
	**Means of transmission of information**		
	Social networks	26	96.3
	SMS	1	3.7
	**Weekly transmission (Monday morning)**		
	Yes	27	100
	No	0	0
	**Involvement of more than half of the staff/speed**		
	Yes	14	51.9
	No	13	48.1
	**Availability of an epidemic management committee for a rapid response**		
	Yes	0	0
	No	27	100
	**Availability of RDT for quick confirmation**		
	Yes	27	100
	No	0	0
	**Drug availability (ACT) for fast care**		
	Yes	27	100
	No		

RDT: rapid diagnostic test

**Reactivity of the epidemiological surveillance system:** overall, the malaria ES system was reactive with a speed of information transmission at the level above 96.3% of the SDP, a harmonized transmission of reports in 100% of the SDP, every Monday morning, an availability of RDTs and drugs for rapid management of suspected and confirmed cases of malaria in all health facilities in the district (Table 3).

### Quality of malaria ES data

**Adequacy of epidemiological surveillance (ES) data:** the total number of malaria cases reported at the district level was not commensurate with the number of cases entered into the DHSI2 platform. A total of 30,846 malaria cases were reported to the district during the year, while 38,457 malaria cases were captured in DHIS2. An error gap of 7611 malaria cases was found and considered under-reported.

**Figure 2:**
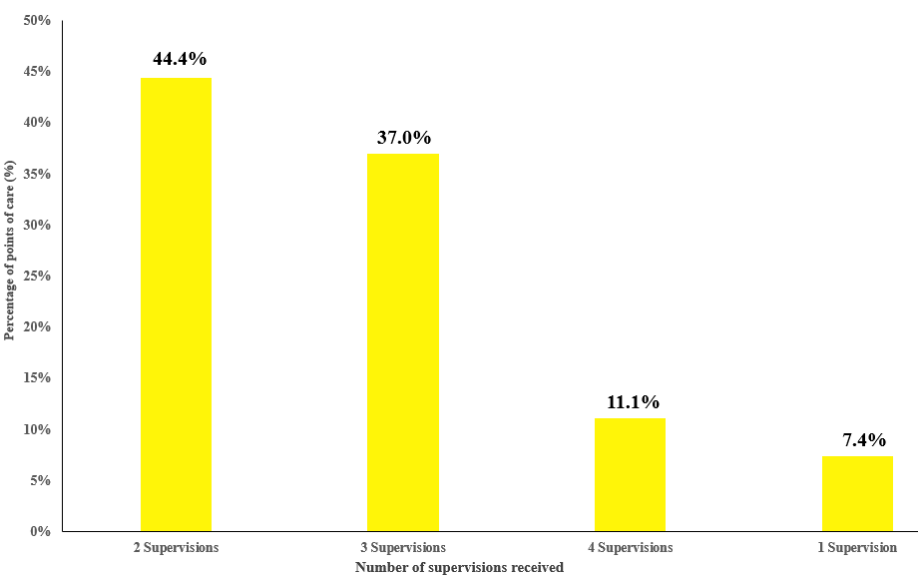
distribution of the number of surveillances received by services delivery points (SDP) on the quality of malaria epidemiological surveillance (ES) data, 2021

**Supervision of SDP on malaria ES data quality:** of the 27 health facilities in our work, only 11.1% received the 4 recommended supervisions during the year on the quality of malaria ES data (Figure 2). However, all structures have received at least one supervision during the year 2021 on malaria.

**Identification of the Strengths, Weaknesses, Opportunities, and Threats (SWOT) of the malaria SE system at the district level:** a consultation of the functioning of the malaria ES system by the providers themselves identified its success and success factors in the form of strengths, weaknesses, opportunities, and threats (Table 4).

**Table 4:** identification of the strengths, weaknesses, opportunities and threats of the malaria epidemiological surveillance system at the services delivery points (SDP) level of the Tambacounda Health District, 2021

Success factors	Failure factors
**Strengths**	**Weaknesses**
Rapid management of suspected and confirmed malaria cases at the SDP level.	Loss of promptness upon correction of the entry after the 15th of the month.
Weekly monitoring of DHIS2 data entry.	Low involvement of private and parapublic SDP in ES activities
Harmonization in the reporting time of cases at the DHIS2 level throughout the district (every Monday morning).	Non-integration of all community data.
Training of 3 district officers in field epidemiology (CMO, DCMO, and FP EPI/ES).	Under-reporting of malaria ES data.
Systematic inclusion of an SE item on the agenda of each district coordination meeting.	Lack of reflexes in analyzing and interpreting malaria ES data by the agents who produce them.
Provision of ES management tools by the district to all SDPs at the beginning of each year (case definitions, notification forms).	Irregularity of supervision at all levels.
Availability of qualified personnel responsible for ES in all SDP.	Single-person management of ES at the SDM level.
	Insufficient number of staff trained in ES and/or epidemic management.
**Opportunities**	**Threats**
Use of social networks (WhatsApp) for tracking ES data and sharing malaria ES guidelines.	Restriction of activities (gatherings and travel) with the context of COVID-19.
Telephone network availability in all SDP for ES data transmission.	Lack of control of travel at the border with The Gambia (porosity of borders).
The existence of several technical and financial partners in the region.	Inaccessibility of some areas of the district during wintering.

## Discussion

It emerged from our study that the malaria ES system of Tambacounda Health District had some strengths but also shortcomings in its attributes which were not favorable to the reduction of morbidity and mortality related to malaria.

We surveyed 27 health facilities with catchment areas covering the entire district. Most of these structures were headed by state-enrolled nursing assistants (44.4%) attesting to the low level of qualification at the head of the SDP. The managers of these structures had considered that the malaria ES system was acceptable despite the fact that the acceptability attribute was subjective. It can, however, be attested by the completeness rate of the reports of the SDP surveyed that were good. This could be related to the filling time of the management tools which was correct according to the heads of health structures. Regular monitoring of malaria data at coordination meetings also contributed to performance against the completeness of the reports. In a study conducted in Nigeria, Visa Tyakaray *et al*. also demonstrated that the country's malaria ES system was acceptable [8]. The same was true of Shaojiin Ma's study of Thailand's malaria epidemiological surveillance system where users of the system felt it was also acceptable [9]. In the study on the documentation of epidemiological surveillance of malaria in the river valley in Senegal and in the suburbs of Dakar, the same results were found with an epidemiological surveillance system for malaria assessed as acceptable [10].

In our work, even if the system was representative with the possibility of illustrating the data in all the health structures surveyed, only 33.3% of them displayed malaria cases on graphs. The possibility of representativeness was mainly related to primary collection tools that were simple to inform and took into account the characteristics of time and place. These results were contrary to those of the Nigeria study where the malaria surveillance system in Kano State collected data that was not representative of all health facilities, as data from two tertiary health facilities and all private facilities were not included in the state's general data [8]. In Kaduna State, Nigeria, work has also shown that the malaria surveillance system excludes data from private health facilities and distorts the comprehensive representation of cases across locations [11]. Thus, in our work, the representativeness of the surveillance system could also be affected by the under-reporting of community data which are not considered in the weekly reports but rather in the monthly morbidity reports of health structures.

Of the 27 SDPs surveyed in our work, nearly three-quarters of those responsible for these structures felt that the malaria ES system was simple. This finding is similar to that of studies conducted in Oyo and Kaduna States, Nigeria, and Chipinge District, Zimbabwe, where surveillance systems were found to be simple and flexible [8,12,13]. However, this contrasts with studies in Brazil, Zimbabwe, and Angola where systems were complex to operate [14,15]. At the Tambacounda Health District level, this simplicity of the malaria ES system was supported by data collection with easily understandable tools and simple reporting to the next level. In addition, the malaria ES system was also flexible with tools adapted to the epidemiological profile of the Tambacounda Health District, which is in a malaria control zone. In all health facilities, the available management tools considered the diagnosis of malaria (with the RDT results columns in the registries) and the possibility of adapting these tools in the event of the introduction of new variables related to ES. These results were like those of Visa Tyakaray *et al*. in Nigeria and those of Chipinge in Zimbabwe and Ebonyi State in Nigeria which also showed that malaria ES systems were flexible and could adapt to changes in malaria data entry [8,13,16].

On the other hand, the stability of the malaria ES system in the Tambacounda District was lacking because in more than half of the SDPs surveyed, as soon as the person in charge of the structure was absent, there was a discontinuity of the ES service. This failure is much more accentuated by the non-involvement of other health workers in surveillance, as the tools were available in all the SDP visits.

In this work, although all health facility managers took care of suspected and/or confirmed cases of malaria and transmitted the information to the next level, the data produced on the spot were not analyzed by the providers themselves. The majority (85.2%) were unaware of the usefulness of epidemiological surveillance for malaria and, in turn, did not use the data to make decisions. When training health workers on ES in general, and during supervision activities, the focus is not on the aspects of analysis and interpretation of data that very often lead to failures in the analysis of data on-site by the providers who produce it. This was not in line with the results of Visa Tyakaray's work which had shown that in Kano State in Nigeria, epidemiological surveillance data were used for decision-making and the formulation of policies that guide the functioning of the surveillance system [8].

Even if the data were not used by the providers themselves, the system in place was very responsive with a speed in the transmission of information to the next level that was done in a harmonized way, and a diagnostic and care device available in all health facilities. This performance could be explained by the reminders made every Monday morning by the district malaria focal point concerning the transmission and entry of data in the platform but also by the periodic sharing of the level of data transmission in the district electronic working group and during the coordination bodies. This speed of transmission of information in a harmonized manner and the regular monitoring by the district were much more supported using electronic means which improves the speed and facilitates access to epidemiological data thus allowing a faster analysis and response [17].

In addition, the regular monitoring of RDTs and artemisinin-based combination therapy (ACTs) through RDT reporting sheets and drug stock sheets had largely contributed to the availability of these inputs in all health structures surveyed for good responsiveness of the malaria ES system.

Despite the good performance in terms of information transmission (96.3% of the SDP in our study), the malaria data transmitted weekly to the district level via the DHIS2 platform, were not consistent with those of morbidity reported monthly in the SDP report. In many health facilities, community data (health boxes and HCP) and private data were left behind in the transmission of malaria ES data, thus explaining the under-reporting in the ES part with a gap of 7,611 malaria cases throughout 2021. Irregular supervision with on-site data verification had certainly exacerbated this data discrepancy between ES and malaria morbidity. In a study conducted in Kaduna State, Nigeria, the results also showed that the malaria surveillance system excluded data from private health facilities [11].

However, there are some limitations to our work. First, it was not representative of all the health districts of Senegal. However, it can be very useful in other contexts, since in Senegal the health districts have the same functioning as the routine epidemiological surveillance system for malaria. In addition, the community component was not also considered.

Finally, this study could also present a desirability bias because, the participants' responses to self-reported questionnaire can be biased to project a better picture of their performance.

## Conclusion

The evaluation of the routine epidemiological surveillance system for malaria at the level of health facilities in the Tambacounda Health District in 2021 showed an acceptable, simple, flexible, representative, and responsive system. However, significant deficiencies were found in the stability, usefulness of the ES system, and the adequacy of ES data with those of malaria morbidity. An increase in the number of qualified staff at the health posts, capacity building of ES staff, and regular supervision of SDP are needed to make the district's malaria epidemiological surveillance system more efficient to contribute to the control of malaria-related morbidity and mortality in Tambacounda. Further studies would be needed to conduct a qualitative evaluation of routine epidemiological surveillance data for malaria in the district to refine the recommendations in this area.
